# Delayed access to emergency obstetrical care among preeclamptic and non-preeclamptic women in Port-Au-Prince, Haiti

**DOI:** 10.1186/s12884-018-1961-4

**Published:** 2018-08-20

**Authors:** Katharine Hutchinson, Malcolm Bryant, Mary Bachman DeSilva, Deborah Price, Lora Sabin, Lindsay Bryson, Roger Jean Charles, Eugene Declercq

**Affiliations:** 1 Médecins Sans Frontières and Boston University School of Public Health, 715 Albany Street, Boston, 02118 USA; 2 Boston University School of Public Health, Boston, USA; 3 University of New England, Portland, USA; 4 Médecins Sans Frontières, Amsterdam, the Netherlands; 5 Médecins Sans Frontières, Addis Ababa, Ethiopia; 6 Medical School of the State University of Haiti, Port-au-Prince, Haiti

**Keywords:** Maternal mortality, Neonatal mortality, Obstetrics, Urban health

## Abstract

**Background:**

The primary objective of this comparative, cross-sectional study was to identify factors affecting delays in accessing emergency obstetric care and clinical consequences of delays among preeclamptic and non-preeclamptic women in Port-au-Prince, Haiti.

**Methods:**

We administered 524 surveys to women admitted to the Médecins Sans Frontières Centre de Référence en Urgences Obstétricales (CRUO) obstetric emergency hospital. Survey questions addressed first (at home), second (transport) and third (health facility) delays; demographic, clinical, and behavioral risk factors for delay; and clinical outcomes for women and infants. Bivariate statistics assessed relationships between preeclampsia status and delay, and between risk factors and delay.

**Results:**

We found longer delays to care for preeclamptic women (mean 14.6 h, SD 27.9 versus non-preeclamptic mean 6.8 h, SD 10.5, *p* < 0.01), primarily attributable to delays before leaving for hospital (mean 13.4 h, SD 30.0 versus non-preeclamptic mean 5.5 h, SD 10.5). Few demographic, clinical, or behavioral factors were associated with care access. Poor outcomes were more likely among preeclamptic women and infants, including intensive care unit admission (10.7%, vs. 0.5% among non-preeclamptic women, *p* < 0.01) and eclampsia (10.7% vs. no cases, *p* < 0.01) for women, and neonatal care unit admission (45.6% vs. 15.4%, *p* < 0.01) and stillbirth (9.9% vs. 0.5%, *p* < 0.01). Longer delays among both groups were not associated with poorer clinical outcomes.

**Conclusion:**

Pregnant women with preeclampsia in Port-au-Prince reported significant delays in accessing emergency obstetric care. This study provides clear evidence that hospital proximity alone does not mitigate the long delays in accessing emergency obstetrical care for Haitian urban, poor women.

## Background

Despite progress in reducing maternal mortality, Haiti still suffers from some of the worst maternal and neonatal health indicators in the Western Hemisphere. Hypertensive disorders of pregnancy, including preeclampsia and eclampsia, are particularly prevalent, [1, 2] and are the leading cause of pregnancy-related death in the region [3]. An understaffed, underfunded health system with few obstetric providers, exacerbated by several large natural disasters in the past decade, makes access to health care difficult. Although most hospitals and obstetric staff are concentrated in the capital city of Port-au-Prince, [4] access to emergency obstetric care is challenging even in this urban context.

Thaddeus and Maine’s Three Delays Model of maternal mortality describes how delays at home prior to deciding to seek care (the first delay); delays in finding and managing transport to a facility (the second delay); and delays in receiving appropriate treatment once at the facility (the third delay) contribute to maternal mortality [5]. The model was conceptualized to address the experiences of rural women, with the original article titled “Too Far to Walk.” Past studies utilizing the Three Delays Model have identified several factors associated with delays in various contexts, including distance to facility, [6–8] cost, [8] educational level, [8] women’s status in the family, [9] perception of the gravity of the situation, [10] transportation options, [11] distribution of facilities, and perception of quality at the facility. [12, 13] Relatively few studies, [14–17] however, have examined how women in urban contexts access emergency care, and the unique delays they encounter. One such study in Kenya examined how access and outcomes among urban poor women may be even worse than their rural counterparts [14]. Urban women who develop obstetric complications, such as preeclampsia and eclampsia, need timely access to high-quality obstetric care to prevent dangerous complications, but often face specific barriers to access that are unique to these settings. The goal of this study was to examine the delays faced by women with preeclampsia while accessing emergency obstetric care in urban Port-au-Prince, the relative importance of the factors associated with delays, and the relationship between those delays and maternal and neonatal health outcomes.

## Methods

### Study setting

Haiti’s public health system suffers from inadequate funding, insufficient trained health staff, and severe shortages of medications and supplies [18]. Demographic and Health Survey (DHS) data indicate that although most pregnant Haitian women accessed antenatal care, the majority reported difficulties accessing care for problems during pregnancy [19].

Médecins Sans Frontières (MSF) operates the Centre de Référence en Urgences Obstétricales (CRUO) emergency obstetric hospital in Port-au-Prince, which admits more than 500 women per month with complications of pregnancy or delivery. CRUO does not offer antenatal care, though women who deliver at the facility are followed up with postpartum visits. More than a quarter of the admitted women have preeclampsia or eclampsia [20]. CRUO is the only large facility in Port-au-Prince offering free obstetric services. Because CRUO is an emergency referral hospital, women with no specific risk factors of pregnancy are generally not admitted, unless delivery is imminent.

### Study procedures

We conducted a study on delays to access emergency obstetric care for preeclamptic and non-preeclamptic pregnant women admitted to the CRUO hospital. This paper reports the quantitative findings from the larger mixed methods study. We utilized a design consisting of a quantitative survey and medical records review to examine delays to care among preeclamptic and non-preeclamptic women; demographic, clinical and behavioral risk factors for delay to care; and the association between delay to care and poor maternal and neonatal clinical consequences.

### Inclusion and exclusion criteria

All women who delivered a baby at CRUO and had a medical diagnosis of preeclampsia or eclampsia, or normal pregnancy, were eligible for the study. CRUO admitting staff determined whether women met the diagnostic criteria. Women who were unconscious and admitted to the Intensive Care Unit and met all inclusion criteria were also included via interviews with their legal representatives. To ensure consistency with the non-preeclamptic group, who all delivered during the hospitalization, the preeclamptic group was also limited to women who delivered. Women admitted to CRUO after delivery for postpartum or gynecologic problems were also ineligible. Non-preeclamptic participants included those women admitted to CRUO with a diagnosis of “physiologic labor” merely because delivery was imminent.

### Measures and data collection methods

The survey recorded self-reported clinical pregnancy history, information about the onset of the symptoms that prompted the decision to leave, how long it took to leave home after the decision to leave was made, the type and cost of transport used, the number and location of other health centers visited prior to CRUO, and the time it took to reach the health center. The time each patient spent in the triage unit prior to admission was recorded from the medical chart.

To ensure that women who died during labor or after delivery were not excluded from the analysis, which could bias study results, women were identified for inclusion at admission to CRUO. Women were identified for inclusion at admission to CRUO, but were not approached for written consent until after delivery and once their medical condition was stable and they were medically able to provide consent and participate. Women were interviewed after delivery, before their discharge from CRUO. The survey was administered to the postpartum patient herself, or in cases of death or severe medical disability, to a legal representative who could give written consent. Study personnel administered surveys verbally in private settings in Haitian Creole or French, depending on the participant’s preference. After survey administration, a medical records review, including maternal and infant outcomes, was completed for each participant by the same study personnel who had completed the survey. These secondary outcomes were recorded from the chart to reduce any reporting bias on the part of the participants, who may have not been able to report medical terminology. Study recruitment and data collection occurred between November 2014 and January 2015.

### Sample size considerations

The sample size was calculated using delay to care as the outcome. The population-based DHS, conducted in 2012, showed that almost 75 % of urban pregnant women in Haiti experienced a difficulty accessing health care during their most recent pregnancy. We expected that women with complications of pregnancy at CRUO had a similar likelihood of delay to care, regardless of whether or not they were diagnosed with preeclampsia. Distance to the nearest health center was found in one study in rural Haiti to be the largest factor in access to prenatal care. We assumed that distance to the hospital would be the most important factor in delay to emergency obstetric care. We also assumed that 20 % of women lived more than an hour away from the hospital, a conservative estimate given the logistical challenges of travel in Port au Prince. Further, it seemed reasonable to presume that: a) this prevalence would likely be similar between women diagnosed with preeclampsia and women with low-risk pregnancies and b) women not exposed to this risk factor (living far away) would have a lower risk of delay, estimated at a 50%.

We measured the exposed group, the women who live far away, by the amount of time they took to reach the hospital, by whichever method they chose to come. Women who took one hour or longer to reach the hospital (whether by walking, public transit, or other means), were categorized as “exposed”. Therefore, the “unexposed” group comprised women whose travel time to the hospital in this instance was less than one hour.

The sample size was calculated to show differences in delayed access to care between the women exposed to a behavioral, demographic or clinical risk factor, and those who did not have a risk factor. Thus, we used the following assumptions for the calculation of the sample size: 1) two-sided significance level of 95%; 2) power of the study 80%; 3) ratio of unexposed/exposed (4:1); 4) expected relative risk of delayed access in the exposed group 1.5; 5) expected proportion of outcome in unexposed group 50%. This generated a sample size calculation of 42 women in the exposed group (women who live far away) and 168 women in the unexposed group (women living close to the hospital), a total of 210 women.

The planned sample size for the survey was 420 women (210 women in each group, preeclamptic and non-preeclamptic), designed to show differences for each group in access to care between women with the primary risk factor (longer distance from the hospital, based on prior literature) and those without the risk factor. Because about 30% of preeclamptic women were not able to report on either the first or second delay due to sickness or eclampsia, an additional 82 preeclamptic women were added, for a total of 292 preeclamptic participants and a grand total of 502 women, assuming a similar rate of non-response regarding first or second delays among the added participants.

### Outcome measures

The primary outcome, total delay, represents the length of time between the onset of labor or complications, and the administration of appropriate treatment at the hospital. If the time was greater than six hours, it was classified as a delay, based on UNFPA recommendations [21]. We recorded delay as a continuous variable so that the association of delay with clinical outcomes could also be investigated as a dose-response relationship. The relationship between risk factors and delay was also evaluated for very long delay (> 24 h), and extremely long delay (> 72 h).

### Analytic methods

We used bivariate analysis to assess the association between delayed access to emergency obstetric care (exposure) and clinical result (outcome). Findings are presented as relative risks with 95% confidence intervals (CI). The analysis also examined relationships between clinical, demographic, and behavioral risk factors and delay. When sample sizes were too small for separate analysis of subgroups (e.g. only 27 women had university education), categories were collapsed. We calculated the association between each individual antecedent risk factor (exposures) and delayed access to health care (outcome). Results were then stratified by preeclampsia status. Statistical analyses were conducted using JMP, Version 11 (SAS Institute Inc., Cary, North Carolina).

The study was approved by the Institutional Review Board of Boston University Medical Center, the Ethics Review Board of Médecins Sans Frontières, and the Haiti Ethics Review Board.

## Results

### Background characteristics of survey respondents

Five hundred and two women were initially enrolled, but twenty-two women were withdrawn because they were unable to complete most survey questions. These women had suffered eclamptic seizures and were unable to remember enough information about the events prior to their hospitalization to answer survey questions.

Background characteristics of the entire sample are presented in Table 1. There were no significant differences in multiparity between the preeclamptic and non-preeclamptic participants. Most women who participated in this study were married, had a religious affiliation, had completed at least primary school education, and were not formally employed. Few study participants (7.3%) self-reported “doing well” or “good” in terms of socioeconomic status.

**Table 1:** Demographic and behavioral characteristics of study participants, by preeclampsia status

	Non-preeclamptic n (%)	Preeclamptic n (%)	*p*-value
	*n* = 210	*n* = 292	
Mean age (SD)	27.2 (6.2)	29.7 (6.8)	< 0.01
Married or cohabitating	149 (71.3)	223 (76.4)	0.20
Attended some secondary school or university	152 (73.1)	206 (71.3)	0.66
Employed in formal sector	15 (7.4)	16 (5.6)	0.43
Had religious affiliation	184 (88.0)	276 (95.2)	< 0.01
Owned home	64 (30.5)	138 (47.3)	< 0.01
Internally displaced	1 (0.5)	8 (2.8)	0.04
“Doing well” or “good” (reported economic status)^1^	12 (5.8)	25 (8.7)	0.22
ANC^2^ with health professional	203 (96.7)	281 (96.2)	0.80
Adequate ANC (4+ visits)	171 (84.2)	231 (83.1)	0.74
Diagnosis of preeclampsia in current pregnancy	15 (7.2)	136 (46.9)	< 0.01
Birth plan made with health professional^3^	191 (91.0)	258 (89.0)	0.35
Birth plan made with TBA	35 (16.6)	38 (13.0)	0.18
ANC with TBA	43 (20.6)	61 (20.9)	0.77
Remembered any preeclampsia danger signs	31 (14.8)	93 (31.9)	< 0.01
Any medications taken in pregnancy	193 (91.9)	266 (91.1)	0.34
Medications prescribed by health professional	191 (91.0)	267 (91.8)	0.20
Antihypertensive medications reported	0 (0)	38 (13.0)	< 0.01
Did not remember the name of medications taken	86 (41.0)	102 (34.9)	0.17

^1^Economic status self-reported via four-item scale using locally appropriate terminology, choices included “doing well”, “good”, “not too bad” and “no money”

^2^ANC = Antenatal care. DHS = Demographic and Health Surveys. TBA = Traditional birth attendant

^3^Birth plans refer to a discussion between provider and patient about intentions and plans for birth

Similar proportions of women who delivered preterm (32–36 weeks gestation) and very preterm (< 32 weeks gestation) reported attending four antenatal care (ANC) visits as did women who delivered at term (84.0% of all women, data not shown). Almost half (46.9%) of preeclamptic women had received a diagnosis of preeclampsia prior to their admission to CRUO, along with 7.2% of women in the non-preeclamptic group. However, less than one third (31.9%) of preeclamptic women could recall any danger sign of worsening preeclampsia, such as headache or facial swelling, although some of the preeclampsia diagnoses may have been made by the referral facility at the time of the labor and birth.

#### Delay to accessing obstetric care

Table 2 summarizes findings relating to the Three Delays [5].

**Table 2:** First, Second, Third, and Total Delay in Preeclamptic and Non-Preeclamptic groups

	Non-Preeclamptic	Preeclamptic	*p*-value
First Delay (hours)	*n* = 207	*n* = 285^a^	
Mean (standard deviation, SD)	5.5 (10.5)	13.4 (30.0)	< 0.01
Median (interquartile range, IQR)	3.0 (5.0)	2.0 (12.0)	
Second Delay (hours)	*n* = 194	*n* = 251	
Mean (SD)	1.0 (0.6)	1.2 (1.0)	0.20
Median (IQR)	1.0 (0)	1.0 (0)	
Third Delay (minutes)	*n* = 208	n = 292	
Mean (SD)	11.6 (38.1)	49.7 (60.2)	< 0.01
Median (IQR)	0 (0)	35.0 (70.0)	
Total Delay (hours)	*n* = 191	*n* = 235	
Mean (SD)	6.8 (10.5)	14.6 (27.9)	< 0.01
Median (IQR)	4 (5.0)	5 (10.6)	

^a^Some participants were unable to recall the length of delays, so not all survey respondents are included in the analysis of each delay

Preeclamptic women experienced a longer mean total delay in accessing emergency obstetrical care than non-preeclamptic women (14.6 h versus 6.8 h, *p* < 0.01). The median total delay was five hours for preeclamptic women, and four hours for non-preeclamptic women (p < 0.01). Longer delays among the preeclamptic group were attributable primarily to waiting longer to leave home (first delay) and a slightly longer third delay. When evaluated as a dichotomous variable (delay > 6 h versus ≤ 6 h), a larger proportion of preeclamptic women reported a long delay compared to non-preeclamptic women (43.8% vs. 33.5%, *p* = 0.03). Almost one-fifth (17.0%) of preeclamptic women reported a delay of twenty-four hours or more before receiving care.

Mean delays for preeclamptic women were longer than median delays due to a portion of cases with extraordinary delays. Twenty-four preeclamptic participants experienced delays longer than 48 h, and for thirteen of those women, the delay was longer than 72 h. An analysis of women with extremely long delays (> 72 h) showed that most had developed preeclampsia earlier in pregnancy. A greater proportion of these women were admitted preterm (84.6% versus 38.6% of all preeclamptic women, *p* < 0.01), and had a low birth weight baby (83.3% versus 46.3% of all preeclamptic women, p < 0.01), than women in the preeclampsia subgroup overall.

### Maternal and neonatal outcomes

More than 10 % of preeclamptic women and almost half of infants experienced poor clinical outcomes. For women, preeclampsia was associated with greater likelihood of cesarean section, intensive care unit admission, coma, eclampsia, and transfusion, compared to non-preeclamptic women (Table 3). Almost one in nine (10.7%) preeclamptic women also experienced an eclamptic seizure. For newborns, a preeclampsia diagnosis for their mothers was associated with greater likelihood of neonatal care unit (NICU) admission, stillbirth, low birth weight (< 2.5 kg), and APGAR score less than 7 at 5 min of life (Table 3). There were 30 total stillbirths in the study, 29 of which occurred to women with preeclampsia.

**Table 3:** Distribution of poor maternal and neonatal outcomes for non-preeclamptic and preeclamptic women

	Non-preeclamptic n (%)	Preeclamptic n (%)	Relative risk associated with preeclampsia exposure (95% CI)	*p-* value
	*n* = 210	*n* = 292		
Mothers				
ICU^a^ admission	1 (0.5)	31 (10.7)	22.4 (3.1–162.6)	< 0.01
Coma	0 (0)	5 (1.7)	–	0.02
Transfusion	1 (0.5)	10 (3.5)	7.3 (0.9–56.4)	0.01
Eclampsia	0 (0)	31 (10.7)	–	< 0.01
Cesarean section	13 (6.2)	190 (65.3)	10.5 (6.2–18.0)	< 0.01
Newborns				
NICU admission^b^	32 (15.4)	131 (45.6)	3.0 (2.1–4.2)	< 0.01
Stillbirth	1 (0.5)	29 (9.9)	20.9 (2.9–151.9)	< 0.01
Low birth weight	31 (15.0)	144 (50.0)	3.3 (2.4–4.7)	< 0.01
APGAR < 7 at 5 min^c^	21 (10.1)	67 (25.6)	2.5 (1.6–4.0)	< 0.01

^a^ICU = Intensive Care Unit

^b^NICU = Neonatal Intensive Care Unit

^c^Excluding stillbirths

### Risks for delay

We found few significant associations between any of the demographic, clinical, or behavioral risk factors and total delay, regardless of whether the delay was measured as binary or continuous. Adequate antenatal care was associated with shorter delays, but only for non-preeclamptic women (data not shown). Living farther from the hospital was not associated with longer delays. In the preeclampsia group, women living in the neighborhood where CRUO is located and the adjacent neighborhood had significantly longer delays (data not shown).

### Risks for poor clinical outcomes by length of delay

Longer total delays were not associated with poorer clinical outcomes (Table 4), except for a greater likelihood of stillbirth among preeclamptic women with shorter delays compared to those with longer delays (14.5% versus 5.9%). Stillbirths were associated with delivering at earlier gestational ages (32 weeks versus 37 weeks, *p* < 0.01, data not shown).

**Table 4:** Maternal and neonatal outcomes by length of delay for preeclamptic and non-preeclamptic women

	Non-Preeclamptic			Preeclamptic		
	Delay < 6 h: n^a^ (%)	Delay ≥ 6 h: n (%)	*p-* value	Delay < 6 h: n (%)	Delay ≥ 6 h: n (%)	*p*-value
Mothers						
ICU admission	1 (0.7)	0 (0.0)	0.45	9 (6.9)	11 (10.9)	0.28
Coma	0 (0.0)	0 (0.0)		1 (0.8)	1 (1.0)	0.86
Transfusion	1 (0.7)	0 (0)	0.45	5 (3.9)	2 (2.0)	0.41
Eclampsia	no cases	no cases		13 (9.9)	9 (9)	0.81
Cesarean section	7 (4.9)	5 (10.4)	0.19	80 (61.1)	66 (65.4)	0.50
Newborns						
NICU admission	18 (12.8)	9 (18.8)	0.32	52 (40.9)	44 (43.6)	0.69
Stillbirth	0 (0.0)	1 (2.1)	0.96	19 (14.5)	6 (5.9)	0.03
Low birth weight (< 2500 g)	22 (15.7)	5 (10.4)	0.35	65 (50.0)	46 (46.0)	0.55
APGAR < 7 at 5 min	12 (8.5)	7 (14.6)	0.24	46 (35.1)	30 (29.7)	0.36

^a^Survey participants who did not answer length of delay were excluded

## Discussion

Urban, poor women in Haiti face dangerous delays to accessing emergency obstetric care. Preeclamptic and eclamptic women in this study experienced a delay in receiving appropriate medical treatment of more than fourteen hours, on average. Median delays were much shorter, reflecting the impact of a subset of women with exceptionally long delays. The vast majority of participants had accessed antenatal care, and yet few could identify any warning signs of preeclampsia. Almost one fifth of preeclamptic participants experienced a twenty-four-hour delay in accessing health care after symptoms started. These very long delays were primarily attributable to long first delays, over thirteen hours after symptoms developed, on average, for this group. Third (triage/health facility) delays were also significantly longer in this group, though that may have been attributable to so many in the non-preeclamptic group arriving at the hospital in advanced labor. Second (transport) delays were comparable in the two groups. Preeclamptic women did not have shorter delays to accessing care, even if they lived relatively close to the hospital. Large proportions of women experienced adverse clinical outcomes, including eclampsia and ICU admission for mothers, and NICU admission and stillbirth for newborns.

Few studies examining the Three Delays have attempted to quantify the length of time for each delay. Our findings are generally consistent with those from comparable prior studies. Researchers in India found an average first delay of eight hours among cases of maternal mortality, compared to a two-hour first delay among controls [7]. Researchers in The Gambia and Malawi found first delays among a minority of cases of maternal death, ranging from two days to a month. [22, 23] A recently published study from Ethiopia found very long delays, averaging more than five days, for women experiencing danger signs of preeclampsia [24].

Although we had expected to identify subgroups of women who were more likely to experience delays, we found no demographic factors associated with longer delays. This lack of association may reflect several possibilities unique to the Haitian context. The vast majority of women in this study were poor and lacked formal employment. Poverty creates an overwhelming barrier to access for all mothers, perhaps rendering other associations to longer delays less important. We found a trend towards shorter delay among preeclamptic women with better economic status (4.9 h versus 15.2 h), although this did not reach statistical significance, likely given the small number of women in our sample (*n* = 25) who reported faring well economically. Economic factors may have similarly limited access for the women in our study [14] even though geographic distances were relatively short.

The study aimed to identify the role of delayed access to care in poor clinical outcomes for mothers or babies. However, the association between long delays to care and poor clinical outcomes was not clearly evident from the data. Difficulties in assessing delay may have led to an under-reporting of delay for preeclamptic women. The study sample size may have been insufficient to find real associations that do exist. Additionally, the generally low economic status of participants and the difficulty they faced in finding health care may have minimized differences between the delayed and not delayed groups. Finally, neonatal death was not able to be accurately identified in this study, due to the inability to track neonatal outcomes after the time of survey administration. However, it is likely that neonatal deaths, even if accurately reported, would have been too rare to permit detection of major differences among the groups.

Long transportation delays were uncommon among both the preeclamptic and non-preeclamptic groups in our study. Prior studies of maternal mortality and the Three Delays Model in a rural context have focused on the distance between women’s homes and delivery facilities as a major factor in both first and second delays [5, 6, 11, 13, 22, 25–27]. The long delays we found among preeclamptic women living relatively close to the hospital may improve understanding of how living short distances from a facility may, somewhat counter-intuitively, delay women’s decisions to leave home. Most women lived within two to five kilometers of a hospital; however, the effects of urban congestion, including traffic and transport difficulties may have contributed to longer delays. Some neighborhoods close to the CRUO hospital routinely experience high levels of violence, and travel may be too dangerous during nighttime hours, which also could have affected delays. However, some studies have found that women’s decisions to leave home for facility birth are unaffected by distance when health facilities are less than five kilometers away [13, 28].

If distance is not a main contributing factor, then what factors determine how urban women and their families make decisions about seeking emergency care? In addition to economic concerns, our study highlights the importance of prioritizing the quality of ANC to identify, treat, refer, and educate higher risk patients such as the preeclamptic mothers studied here, who reported little understanding of danger signs and the importance of seeking care. A lack of understanding of the gravity of their symptoms might explain many women’s long first delay at home prior to leaving to seek care. Other studies have found similarly low levels of knowledge of preeclampsia danger signs, [24, 29–31] which contributed to delayed access to care [10, 23, 14]. Despite reporting at least four antenatal visits and a hypertension diagnosis, most preeclamptic women in this study were unaware of the specific symptoms that should prompt immediate medical attention. Given this lack of knowledge among the study population, our study identifies needs for improvement in ANC practices in Haiti and potentially in other low resource urban settings.

The measurement of delay represented the most important study limitation. As with all self-reported data, it is unclear to what extent study participants reported delays accurately. Although the interviewers attempted to clarify responses with participants, as well as to triangulate information with family members, it is possible that reported delays were not accurate. There were also challenges for participants in estimating the first delay. The onset of the first delay corresponds to the onset of the emergency, which is subjective and specific to the experience of each participant. For women without preeclampsia, who were in labor, the onset of the first delay typically corresponded with the onset of strong contractions, or when their water broke. Moreover, the non-preeclamptic women in our study were not an ideal comparison group as they were a select group of women in spontaneous labor who were admitted just prior to delivery. Fifteen participants in the normal group also stated that they had been diagnosed with preeclampsia during that pregnancy, and some of them had elevated blood pressure readings on admission. If these women were on the verge of delivering their infant, it is possible that staff did not have the time to distinguish between blood pressures that can be normally elevated during delivery, and those indicating preeclampsia. Misclassification of women by preeclampsia diagnosis could have biased the delays reported by these groups, by artificially lengthening delays in the normal group. It is unlikely that a misclassification of these fifteen participants would have affected the study outcomes. The non-preeclamptic women had shorter delays partly since they were, as a group, closer to delivery than their preeclamptic counterparts. Preeclamptic women’s lack of knowledge of danger signs meant that they were unable to identify the start of the delay, possibly leading to reporting artificially shorter delays.

## Conclusion

In the more than twenty years since the seminal study proposing the Three Delays Model, the model has guided critical efforts to reduce maternal and neonatal mortality. Refining this model to include urban women, and the unique delays they face, will serve to include the essential experiences of these women who also sometimes face long delays to care. A renewed focus on the accessibility and quality of maternal health services, including newly published WHO guidelines on antenatal care, [32] provides an opportunity to improve quality and accessibility of these lifesaving services. This study reinforces the urgent need to ensure that urban poor women are included—and prioritized—in these efforts.

## Abbreviations

ANC: Antenatal care
CRUO: Centre de Référence en Urgences Obstétricales (Obstetric Emergency Referral Center)
DHS: Demographic and Health Surveys
ICU: Intensive Care Unit
MSF: Médecins Sans Frontières (Doctors Without Borders)
NICU: Neonatal Intensive Care Unit
TBA: Traditional birth attendant
UNFPA: United Nations Population Fund
WHO: World Health Organization
